# Joint effect of pre-operative anemia and perioperative blood transfusion on outcomes of colon-cancer patients undergoing colectomy

**DOI:** 10.1093/gastro/goz033

**Published:** 2019-08-09

**Authors:** Zheng Liu, Jia-Jun Luo, Kevin Y Pei, Sajid A Khan, Xiao-Xu Wang, Zhi-Xun Zhao, Ming Yang, Caroline H Johnson, Xi-Shan Wang, Yawei Zhang

**Affiliations:** 1 Department of Colorectal Surgery, National Cancer Center/National Clinical Research Center for Cancer/Cancer Hospital, Chinese Academy of Medical Sciences and Peking Union Medical College, Beijing, China; 2 Department of Surgery, Yale School of Medicine, New Haven, CT, USA; 3 Department of Surgery, School of Medicine, Texas Tech University Health Sciences Center, Lubbock, TX, USA; 4 Department of Environmental Health Sciences, Yale School of Public Health, New Haven, CT, USA

**Keywords:** colon cancer, colectomy, anemia, blood transfusion

## Abstract

**Background:**

Both pre-operative anemia and perioperative (intra- and/or post-operative) blood transfusion have been reported to increase post-operative complications in patients with colon cancer undergoing colectomy. However, their joint effect has not been investigated. The purpose of this study was to evaluate the joint effect of pre-operative anemia and perioperative blood transfusion on the post-operative outcome of colon-cancer patients after colectomy.

**Methods:**

We identified patients from the American College of Surgeons National Surgical Quality Improvement Program (NSQIP) database 2006–2016 who underwent colectomy for colon cancer. Multivariate logistic regression analysis was employed to assess the independent and joint effects of anemia and blood transfusion on patient outcomes.

**Results:**

A total of 35,863 patients—18,936 (52.8%) with left-side colon cancer (LCC) and 16,927 (47.2%) with right-side colon cancer (RCC)—were identified. RCC patients were more likely to have mild anemia (62.7%) and severe anemia (2.9%) than LCC patients (40.2% mild anemia and 1.4% severe anemia). A total of 2,661 (7.4%) of all patients (1,079 [5.7%] with LCC and 1,582 [9.3%] with RCC) received a perioperative blood transfusion. Overall, the occurrence rates of complications were comparable between LCC and RCC patients (odds ratio [OR]* *=* *1.01; 95% confidence interval [CI]* *=* *0.95–1.07; *P *=* *0.750). There were significant joint effects of anemia and transfusion on complications and the 30-day death rate (*P* for interaction: 0.010). Patients without anemia who received a transfusion had a higher risk of any complications (LCC, OR* *=* *3.51; 95% CI* *=* *2.55–4.85; *P *<* *0.001; RCC, OR* *=* *3.74; 95% CI* *=* *2.50–5.59; *P *<* *0.001), minor complications (LCC, OR* *=* *2.54; 95% CI* *=* *1.63–3.97; *P *<* *0.001; RCC, OR* *=* *2.27; 95% CI* *=* *1.24–4.15; *P *=* *0.008), and major complications (LCC, OR* *=* *5.31; 95% CI* *=* *3.68–7.64; *P *<* *0.001; RCC, OR* *=* *5.64; 95% CI* *=* *3.61–8.79; *P *<* *0.001), and had an increased 30-day death rate (LCC, OR* *=* *6.97; 95% CI* *=* *3.07–15.80; *P *<* *0.001; RCC, OR* *=* *4.91; 95% CI* *=* *1.88–12.85; *P *=* *0.001) than patients without anemia who did not receive a transfusion.

**Conclusions:**

Pre-operative anemia and perioperative transfusion are associated with an increased risk of post-operative complications and increased death rate in colon-cancer patients undergoing colectomy.

## Introduction

Colon cancer is one of the most common malignancies worldwide [1]. In the USA, it was estimated that 97,220 new colon-cancer cases would be diagnosed in 2018 [2]. Surgical resection is the only curative treatment modality for colon cancer; mortality should be kept low with adequate assessment of comorbidities [3]. A large number of colon-cancer patients have anemia [4, 5] and pre-operative anemia has been reported to be associated with poor post-operative outcomes in patients undergoing colectomy [6].

Perioperative (intra- and/or post-operative) blood transfusion is sometimes necessary for colon-cancer patients with anemia who underwent surgery [7, 8]. However, the relationship between blood transfusion and post-operative outcomes is unclear, as published studies report conflicting results [9–12]. Although there are some suggestions that transfusions and pre-operative anemia are both independent predictors of a poor outcome [6, 13], no studies have investigated the joint effect of anemia and transfusion on surgical outcomes among colon-cancer patients who have undergone colectomy. Transfusions are commonly perioperatively given to patients with anemia during surgery [14]. Due to the strong association between anemia and transfusion, the joint effect of anemia and transfusions should be considered. Additionally, there is growing evidence that demonstrates that right colon cancer (RCC) and left colon cancer (LCC) might be different malignancies [15–18]. It is not clear whether anemia and transfusions have differential effects on RCC or LCC outcomes. Furthermore, stratification by tumor location provides an opportunity to identify patients who may benefit from a transfusion treatment. This study aimed to evaluate the combined effect of anemia and transfusion on surgical outcomes stratified by tumor location.

## Material and methods

### Data source and study population

The American College of Surgeons National Surgical Outcome Improvement Program (ACS NSQIP) database is a nationally validated, risk-adjusted, outcomes-based program used to measure and improve the quality of surgical care. A total of 39,729 patients who underwent elective laparoscopic or open colectomy for RCC or LCC were identified from the NSQIP 2006–2016 database. RCC colectomy was defined as a partial colectomy with ileocolic anastomosis (current procedural terminology [CPT] codes: 44160 or 44205) for a malignant neoplasm of the colon (the *International Classification of Diseases, Ninth Revision* [*ICD-9*] or *Tenth Revision* [*ICD-10*]), cecum (153.4 or C18.0), ascending colon (153.6 or C18.2), or hepatic flexure (153.0 or C18.3). LCC colectomy was defined as a partial colectomy (CPT codes: 44140, 44204, 44145, or 44207) for malignant neoplasm of the descending colon (153.2 or C18.6) or sigmoid colon (153.3 or C18.7).

Patients admitted as urgent or emergency cases; patients with missing information on hematocrit analysis or blood-transfusion details; patients with sepsis, open wounds, or wound infections; and patients dependent on a ventilator were excluded from consideration. This study was designed to compare outcomes between LCC and RCC, so the patients undergoing total colectomy were excluded.

### Variable definition

Using the pre-operative hematocrit levels, anemia was categorized as severe (hematocrit <26%), moderate (26% to <30%), mild (30% to <38%), and no anemia (≥38%) according to the criteria established in a previous study [6]. We combined the moderate and mild anemia categories into the ‘mild anemia’ group according to a previous study [19]. Perioperative blood transfusion was defined as patients who received a transfusion any time from the start of surgery to 72 hours after surgery (intra- and/or post-operative). Minor complications included superficial surgical-site infection (SSI), urinary-tract infection (UTI), deep venous thrombosis (DVT), and thrombophlebitis. Major complications included deep SSI, organ-space SSI, wound disruption, pneumonia, re-intubation, pulmonary embolism, greater than 48-hour post-operative ventilator-assisted respiration, progressive renal insufficiency, acute renal failure (ARF), cardiovascular accident (CVA), cardiac arrest requiring cardiopulmonary resuscitation, myocardial infarction (MI), sepsis, and septic shock. Patients experiencing any complication were defined as having at least one minor or major complication.

### Statistical analysis

The baseline characteristics between RCC and LCC patients were compared using Pearson’s chi-square tests for categorical variables and Student’s *t*-test for continuous variables. Multivariate logistic regression models were employed to examine the associations of surgical outcomes with anemia and transfusion while adjusting for potential confounding variables. The significance of the anemia–transfusion interaction was assessed by adding an interaction term in the logistic regression models. All statistical tests were two-sided. A *P *<* *0.05 was considered statistically significant. All statistical analyses were performed using SAS (version 9.4, SAS Institute Inc, Cary, NC, USA).

## Results

### Demographics and comorbidities

After applying the exclusion criteria, 35,863 patients (18,936 LCC and 16,927 RCC) were analysed. Compared with RCC patients, LCC patients were more likely to be younger, male, non-white, and current smokers **(**Table 1**)**, and were also more likely to be functionally independent and have an American Society of Anesthesiologists (ASA) classification of <3 and a body mass index (BMI) of >30 kg/m². LCC patients experienced less weight loss and comorbidities of diabetes mellitus, MI, congestive heart failure (CHF), previous cardiac surgery, previous percutaneous coronary intervention (PCI), transient ischemic attack (TIA), hypertension, pneumonia, chronic obstructive pulmonary disease (COPD), CVA, and hemiplegia compared with RCC patients. No significant differences were observed in surgery approach and comorbidities of revascularization or amputation, renal failure, dialysis, impaired sensorium, or quadriplegia. The percentages of RCC patients who had mild anemia (62.7% vs 40.2%, respectively; *P *<* *0.001) and severe anemia (2.9% vs 1.4%, respectively; *P *<* *0.001) were lower than those of LCC patients. A total of 2,661 (7.4%) of all patients (1,079 [5.7%] with LCC and 1,582 [9.3%] with RCC) received a perioperative transfusion.

**Table 1.** Distribution of 35,863 patients with colon cancer from the American College of Surgeons National Surgical Outcome Improvement Program (ACS NSQIP) database

Characteristic	No. of patients (%)		*P*-value
	LCC (*n* = 18,936)	RCC (*n* = 16,927)	
Age, years			
≤49	2,541 (13.4)	971 (5.7)	<0.001
50–64	7,303 (38.6)	4,100 (24.2)	
65–79	6,627 (35.0)	7,233 (42.7)	
≥80	2,465 (13.0)	4,623 (27.3)	
Sex			
Female	8,841 (46.7)	9,291 (54.9)	<0.001
Male	10,095 (53.3)	7,636 (45.1)	
Race			
White	12,178 (64.3)	11,822 (69.8)	<0.001
Black	1,680 (8.9)	1,596 (9.4)	
Others	5,078 (26.8)	3,509 (20.7)	
Current smoker			
No	16,508 (87.2)	14,948 (88.3)	0.001
Yes	2,428 (12.8)	1,979 (11.7)	
Functional status			
Independent	18,503 (97.7)	16,266 (96.1)	
Partially or fully dependent	433 (2.3)	661 (3.9)	<0.001
ASA classification			
<3	9,050 (47.8)	6,074 (35.9)	<0.001
≥3	9,886 (52.2)	10,853 (64.1)	
BMI, kg/m²			
<18.5	340 (1.8)	393 (2.3)	<0.001
18.5–24.9	4,659 (24.6)	4,511 (26.7)	
25–29.9	6,454 (34.1)	5,784 (34.2)	
≥30	7,483 (39.5)	6,239 (36.9)	
Weight loss >10%			
No	18,168 (95.9)	16,000 (94.5)	<0.001
Yes	768 (4.1)	927 (5.5)	
Surgery procedure			
Laparoscopic	11,330 (59.8)	10,005 (59.1)	0.19
Open	7,606 (40.2)	6,922 (40.9)	
Comorbidities			
Diabetes mellitus	3,499 (18.5)	3,474 (20.5)	<0.001
MI	35 (0.2)	61 (0.4)	0.001
CHF	154 (0.8)	238 (1.4)	<0.001
Revascularization or amputation	78 (0.4)	68 (0.4)	0.88
Previous cardiac surgery	335 (1.8)	410 (2.4)	<0.001
Previous PCI	378 (2.0)	403 (2.4)	0.01
TIA	150 (0.8)	197 (1.2)	<0.001
Hypertension	10,025 (52.9)	10,276 (60.7)	<0.001
Pneumonia	1 (0)	13 (0.1)	<0.001
COPD	847 (4.5)	1, 112 (6.6)	<0.001
Renal failure	66 (0.4)	66 (0.4)	0.52
Dialysis	100 (0.5)	98 (0.6)	0.52
CVA	110 (0.6)	147 (0.9)	0.001
Impaired sensorium	5 (0)	5 (0)	0.86
Hemiplegia	38 (0.2)	64 (0.4)	0.002
Quadriplegia	4 (0)	2 (0)	0.5
Operation approach			
Open	7,606 (40.2)	6,922 (40.9)	0.1619
Laparoscopic	11,330 (59.8)	10,005 (59.1)	
Anemia			
No	11,073 (58.5)	5,829 (34.4)	
Mild	7,602 (40.2)	10,606 (62.7)	<0.001
Severe	261 (1.4)	492 (2.9)	<0.001
Blood transfusion			
No	17,867 (94.4)	15,345 (90.7)	<0.001
Yes	1,069 (5.7)	1,582 (9.4)	

LCC, left colon cancer; RCC, right colon cancer; ASA, American Society of Anesthesiologists; BMI, body mass index (calculated as weight in kilograms divided by height in meters squared); MI, myocardial infarction; CHF, congestive heart failure; PCI, percutaneous coronary intervention; TIA, transient ischemic attack; COPD, chronic obstructive pulmonary disease; CVA, cerebrovascular accident.

### Association between outcomes and locations

After adjusting for patient demographics and comorbidities, there was no significant difference in complications between RCC and LCC patients **(**Table 2**)**. When specific complications were considered, RCC patients had higher risk of pneumonia (odds ratio [OR]* *=* *1.23; 95% confidence interval [CI]* *=* *1.04–1.45; *P *=* *0.016), re-intubation (OR* *=* *1.04; 95% CI* *=* *1.04–1.04; *P *<* *0.001), and DVT (OR* *=* *1.28; 95% CI* *=* *1.02–1.60; *P *=* *0.033) than LCC patients. RCC patients had lower risk of wound disruption (OR* *=* *0.69; 95% CI* *=* *0.54–0.89; *P *=* *0.003), ventilator dependence (OR* *=* *0.89; 95% CI* *=* *0.89–0.89; *P *<* *0.001), and ARF (OR* *=* *0.94; 95% CI* *=* *0.94–0.95; *P *<* *0.001) than LCC patients. Lastly, the operation time of colectomy for RCC was shorter than that for LCC patients (OR* *=* *0.38; 95% CI* *=* *0.36–0.39; *P *<* *0.001); however, RCC patients stayed in the hospital longer (OR* *=* *1.10; 95% CI* *=* *1.05–1.15; *P *<* *0.001) than LCC patients.

**Table 2.** Association between surgical outcomes and colon-cancer locations

Outcome	No. of patients (%)		OR^a^ (95% CI)	*P*-value
	LCC (*n* = 18,936)	RCC (*n* = 16,927)		
No complication	16,315 (86.2)	14,238 (84.1)	1 [Reference]	
Any complication^b^	2,621 (13.8)	2,689 (15.9)	1.01 (0.95–1.07)	0.750
Minor complications^c^	1,434 (7.6)	1,369 (8.1)	0.98 (0.90–1.06)	0.642
Major complications^d^	1,419 (7.5)	1,503 (8.9)	1.00 (0.92–1.08)	1.000
Superficial SSI	994 (5.3)	848 (5.0)	0.95 (0.86–1.05)	0.312
Deep SSI	132 (0.7)	119 (0.7)	0.96 (0.74–1.24)	0.759
Organ-specific SSI	498 (2.6)	479 (2.8)	1.08 (0.94–1.23)	0.277
Wound disruption	165 (0.9)	116 (0.7)	0.69 (0.54–0.89)	0.003
Pneumonia	273 (1.4)	390 (2.3)	1.23 (1.04–1.45)	0.016
Re-intubation	246 (1.3)	321 (1.9)	1.04 (1.04–1.04)	<0.001
Pulmonary embolism	97 (0.5)	135 (0.8)	1.25 (0.95–1.65)	0.111
Ventilator dependence	213 (1.1)	216 (1.3)	0.89 (0.89–0.89)	<0.001
Renal progressive insufficiency	102 (0.5)	100 (0.6)	0.91 (0.68–1.22)	0.526
ARF	66 (0.4)	66 (0.4)	0.94 (0.94–0.95)	<0.001
CVA	45 (0.2)	70 (0.4)	1.14 (0.77–1.69)	0.513
Coma	5 (0)	0 (0)	NA	
Cardiac arrest	76 (0.4)	94 (0.6)	1.04 (0.76–1.43)	0.806
MI	115 (0.6)	141 (0.8)	0.94 (0.73–1.22)	0.631
Sepsis	405 (2.1)	406 (2.4)	1.06 (0.91–1.23)	0.454
Septic shock	212 (1.1)	222 (1.3)	0.95 (0.78–1.16)	0.610
UTI	359 (1.9)	393 (2.3)	0.91 (0.78–1.06)	0.230
DVT	150 (0.8)	209 (1.2)	1.28 (1.02–1.60)	0.033
Re-operation	56 (0.3)	41 (0.2)	0.77 (0.50–1.18)	0.235
30-day mortality	175 (0.9)	251 (1.5)	1.04 (0.85–1.27)	0.703
Operation time of colectomy^e^, min				
<160	8,955 (47.3)	12,107 (71.5)	1 [Reference]	
≥160	9,981 (52.7)	4,820 (28.5)	0.38 (0.36–0.39)	<0.001
Length of hospital stay^e^, days				
<5	8,740 (46.2)	6,945 (41.0)	1 [Reference]	
≥5	10,196 (53.8)	9,982 (59.0)	1.10 (1.05–1.15)	<0.001

LCC, left colon cancer; RCC, right colon cancer; OR, odds ratio; CI, confidential interval; SSI, surgical-site infection; ARF, acute renal failure, CVA, cerebrovascular accident; MI, myocardial infraction; UTI, urinary-tract infection; DVT, deep vein thrombosis; NA, not applicable.

^a^ Adjusted for age, sex, race, smoking, functional status, American Society of Anesthesiologists classification, body mass index, weight loss >10%, diabetes, congestive heart failure, previous cardiac surgery, previous percutaneous coronary intervention, myocardial infraction, transient ischemic attack, hypertension, pneumonia, chronic obstructive pulmonary disease, cerebrovascular accident, and hemiplegia.

^b^ Including one or more of the complications listed in Table 2.

^c^ Including superficial SSI, UTI, DVT, and/or thrombophlebitis.

^d^ Including deep SSI, organ-space SSI, wound disruption, pneumonia, re-intubation, pulmonary embolism, greater than 48-hour post-operative ventilator-assisted respiration, progressive renal insufficiency, ARF, CVA, cardiac arrest requiring cardiopulmonary resuscitation, MI, sepsis, and septic shock.

^e^ Median value was based on the distribution of patients with LCC colectomy.

### Association between outcomes and locations according to anemia and blood transfusion

Both anemia and transfusion were independently associated with an increased risk of post-operative complications and death based on multivariate analyses **(**Table 3**)**. The observed increased risks of complications seemed greater among LCC than RCC patients. We also found significant joint effects of anemia and transfusion on complications and 30-day mortality. Among LCC patients without anemia, patients receiving transfusions had higher risk of any complications (OR* *=* *3.51; 95% CI* *=* *2.55–4.85; *P *<* *0.001), minor complications (OR* *=* *2.54; 95% CI* *=* *1.63–3.97; *P *<* *0.001), and major complications (OR* *=* *5.31; 95% CI* *=* *3.68–7.64; *P *<* *0.001) than those receiving transfusions **(**Table 3**)**. The high risks of any complications, minor complications, and major complications were also observed in patients with mild and severe anemia who underwent a transfusion. Similar patterns were observed for RCC patients (OR* *=* *3.74; 95% CI* *=* *2.50–5.59; *P *<* *0.001 for any complication; OR* *=* *2.27; 95% CI* *=* *1.24–4.15; *P *=* *0.008 for minor complications; OR* *=* *5.64; 95% CI* *=* *3.61–8.79; *P *<* *0.001 for major complications) (Table 3). The risk of 30-day mortality was the highest among LCC patients without anemia but who had a transfusion (OR* *=* *6.97; 95% CI* *=* *3.07–15.80; *P *<* *0.001), followed by patients with mild anemia and transfusion (OR* *=* *4.48; 95% CI* *=* *2.75–7.27; *P *<* *0.001). Similarly, in RCC patients, the risk of 30-day mortality was the highest among patients without anemia but with transfusion (OR* *=* *4.91; 95% CI* *=* *1.88–12.85; *P *=* *0.001), followed by patients with severe anemia but no transfusion (OR* *=* *3.18; 95% CI* *=* *1.54–6.57; *P *=* *0.002) and patients with mild anemia and transfusion (OR* *=* *3.10; 95% CI* *=* *2.00–4.81; *P *<* *0.001). Similar associations between anemia/transfusion and overall outcomes were found between open and laparoscopic groups (Supplementary Table 1) and between patients who had longer and shorter operation times (Supplementary Table 2), and all models fit well (Supplementary Table 3).

**Table 3.** Association between surgical outcomes and colon-cancer locations according to anemia and blood transfusion

Variable	No complications	Any complication^b^			Minor complications^c^			Major complications^d^			30-day mortality		
	No. of cases	No. of cases	OR^a^ (95% CI)	*P*-value	No. of cases	OR^a^ (95% CI)	*P*-value	No. of cases	OR^a^ (95% CI)	*P*-value	No. of cases	OR^a^ (95% CI)	*P*-value
LCC													
Anemia													
No	9,794	1, 279	1 [Reference]		728	1 [Reference]		634	1 [Reference]		59	1 [Reference]	
Mild	6,318	1, 284	1.26 (1.15–1.38)	<0.001	680	1.22 (1.08–1.37)	0.001	744	1.41 (1.24–1.59)	<0.001	113	1.55 (1.09–2.21)	0.015
Severe	203	58	1.27 (0.92–1.74)	0.146	26	1.19 (0.77–1.84)	0.433	41	1.54 (1.06–2.24)	0.023	3	0.76 (0.23–2.54)	0.653
Transfusion													
No	15,567	2, 300	1 [Reference]		1, 312	1 [Reference]		1, 184	1 [Reference]		136	1 [Reference]	
Yes	748	321	2.40 (2.06–2.78)	<0.001	122	1.66 (1.34–2.04)	<0.001	235	3.22 (2.71–3.82)	<0.001	39	3.05 (2.05–4.51)	<0.001
Anemia/transfusion													
No anemia, no transfusion	9,662	1, 221	1 [Reference]		704	1 [Reference]		592	1 [Reference]		52	1 [Reference]	
No anemia, transfusion	132	58	3.51 (2.55–4.85)	<0.001	24	2.54 (1.63–3.97)	<0.001	42	5.31 (3.68–7.64)	<0.001	7	6.97 (3.07–15.80)	<0.001
Mild anemia, no transfusion	5,779	1, 049	1.29 (1.17–1.41)	<0.001	590	1.24 (1.09–1.40)	0.001	573	1.46 (1.29–1.66)	<0.001	83	1.74 (1.20–2.52)	0.003
Mild anemia, transfusion	539	235	2.89 (2.43–3.44)	<0.001	90	1.96 (1.53–2.50)	<0.001	171	4.32 (3.52–5.29)	<0.001	30	4.48 (2.75–7.27)	<0.001
Severe anemia, no transfusion	126	30	1.62 (1.07–2.44)	0.023	18	1.67 (1.00–2.77)	0.050	19	2.13 (1.30–3.51)	0.003	1	0.86 (0.12–6.33)	0.881
Severe anemia, transfusion	77	28	2.42 (1.55–3.78)	<0.001	8	1.20 (0.57–2.51)	0.631	22	3.90 (2.38–6.39)	<0.001	2	2.40 (0.57–10.20)	0.233
*P*-value for interaction			0.002			0.006			<0.001			0.01	
RCC													
Anemia													
No	5,042	787	1 [Reference]		434	1 [Reference]		415	1 [Reference]		47	1 [Reference]	
Mild	8,788	1, 818	1.13 (1.02–1.24)	0.019	891	1.04 (0.92–1.18)	0.531	1, 033	1.16 (1.02–1.32)	0.024	190	1.38 (0.98–1.94)	0.065
Severe	408	84	0.87 (0.67–1.13)	0.296	44	0.93 (0.66–1.31)	0.678	55	0.93 (0.68–1.29)	0.650	14	1.55 (0.81–2.97)	0.186
Transfusion													
No	13,078	2, 267	1 [Reference]		1, 192	1 [Reference]		1, 217	1 [Reference]		196	1 [Reference]	
Yes	1,160	422	1.97 (1.73–2.24)	<0.001	177	1.63 (1.37–1.95)	<0.001	286	2.43 (2.08–2.82)	<0.001	55	2.01 (1.46–2.79)	<0.001
Anemia/transfusion													
No anemia, no transfusion	4,972	747	1 [Reference]		421	1 [Reference]		384	1 [Reference]		42	1 [Reference]	
No anemia, transfusion	70	40	3.74 (2.50–5.59)	<0.001	13	2.27 (1.24–4.15)	0.008	31	5.64 (3.61–8.79)	<0.001	5	4.91 (1.88–12.85)	0.001
Mild anemia, no transfusion	7,866	1, 477	1.16 (1.05–1.28)	0.004	744	1.04 (0.91–1.19)	0.565	806	1.22 (1.07–1.39)	0.003	144	1.45 (1.02–2.08)	0.038
Mild anemia, transfusion	922	341	2.21 (1.89–2.57)	<0.001	147	1.74 (1.42–2.14)	<0.001	227	2.79 (2.32–3.37)	<0.001	46	3.10 (2.00–4.81)	<0.001
Severe anemia, no transfusion	240	43	1.09 (0.77–1.52)	0.627	27	1.23 (0.81–1.86)	0.331	27	1.29 (0.85–1.96)	0.232	10	3.18 (1.54–6.57)	0.002
Severe anemia, transfusion	168	41	1.43 (1.00–2.04)	0.050	17	1.09 (0.65–1.82)	0.744	28	1.85 (1.21–2.82)	0.005	4	1.48 (0.52–4.24)	0.463
*P*-value for interaction			<0.001			0.03			<0.001			0.004	

LCC, left colon cancer; RCC, right colon cancer; OR, odds ratio; CI, confidential interval.

^a^ Adjusted for age, sex, race, smoking, functional status, American Society of Anesthesiologists classification, body mass index, weight loss >10%, diabetes, congestive heart failure, previous cardiac surgery, previous percutaneous coronary intervention, transient ischemic attack, hypertension, pneumonia, myocardial infraction, chronic obstructive pulmonary disease, cerebrovascular accident, and hemiplegia.

^b^ Including one or more of the complications listed in Table 2.

^c^ Including superficial surgical-site infection, deep vein thrombosis, urinary-tract infection, and/or thrombophlebitis.

^d^ Including deep surgical-site infection, organ-space surgical-site infection, wound disruption, pneumonia, re-intubation, pulmonary embolism, greater than 48-hour post-operative ventilator-assisted respiration, progressive renal insufficiency, acute renal failure, cerebrovascular accident, cardiac arrest requiring cardiopulmonary resuscitation, myocardial infraction, sepsis, and septic shock.

## Discussion

This study investigated the joint effect of pre-operative anemia and perioperative transfusion by tumor location on post-operative outcomes in colon-cancer patients undergoing colectomy. A novel finding from the study was that patients without anemia but with transfusion experienced the highest risk of complications and mortality, followed by patients with mild or severe anemia and with blood transfusion, suggesting that a conservative transfusion practice should be considered during colectomy.

The exact mechanisms linking transfusion and adverse outcomes are not fully understood; several phenomena have been suggested [20]. For example, transfusion induces immunosuppression, which results in an increased susceptibility to infections [21, 22]. Transfusion can also increase inflammation by inducing alloimmunization [23]. As expected, this study demonstrated that perioperative transfusion was associated with increased risk of post-operative complications and mortality regardless of anemia status in patients who underwent colectomy. Although pre-operative anemia was reported as an independent predictor on post-operative outcomes, the combined effect appears more closely related to blood transfusion. An earlier study based on NSQIP data reported an increased risk of complications and mortality associated with pre-operative transfusion in patients who underwent colectomy [13]. To eliminate potential confounding from pre-operative transfusion, our study population excluded patients with a history of pre-operative transfusion.

Our study found that the risk of post-operative complications associated with transfusion varied by anemia status. There are reasons for transfusion in clinical practice. For example, patients with significant blood loss during or after colectomy [24], patients with certain conditions (liver disease, etc.) affecting the production of clotting proteins [25], patients who underwent chemotherapy, and/or patients with certain heart or lung diseases [26, 27] were all transfusion candidates. It is also possible that the underlying clinical reasons for transfusion resulted in adverse outcomes rather than the transfusion itself. Due to a lack of information on the reason for blood transfusion in our patient cohort, we were unable to evaluate this association. Furthermore, as the decision to transfuse a patient depends on an individual evaluation by a physician, the decision is also influenced by regulations, fear of future litigation, and public expectations in addition to clinical evidence [28]. On the other hand, conditions that suggest a blood transfusion might further worsen of blood transfusion’s adverse effect on outcomes, even after adjusting demographics and comorbidities.

Our study’s findings provide a strong argument for a more optimal and prudent transfusion practice, suggesting a restrictive rather than liberal transfusion strategy [29, 30]. Alternatives to transfusion have been long anticipated. Pre-operative ferric carboxymaltose treatment has been shown to significantly reduce transfusion requirements and hospital length of stay in colon-cancer patients with anemia and improves hemoglobin response at 12 weeks in patients who underwent gastrectomy, suggesting that it could be a viable alternative to transfusion when a rapid increase in the hemoglobin level is required [31, 32].

In addition to providing strong evidence for the joint effect of pre-operative anemia and perioperative transfusion on post-operative outcomes of colectomy, the present study also raised a concern about the transfusion criteria for patients undergoing colectomy for cancer, specifically for patients with no anemia. The most common reason for transfusion in such patients is either large amounts of blood loss during surgery or major bleeding complications after surgery. Nevertheless, this reason cannot be explained by the NSQIP, which does not collect any data on blood loss.

A common concern is that case complexity is associated with transfusion requirements. Without a reliable measurement of case complexity, the operation time could serve as a surrogate indicator. Further, laparoscopic surgery was also associated with lower transfusion requirements than an open approach [8] likely because open surgery may amplify the effect of anemia and blood transfusion. In comparison, we found that patients who experienced open surgery or longer operation time did not show different patterns.

Another interesting finding was the stronger association of anemia and transfusion with complications and mortality in LCC patients than in RCC patients. To our knowledge, this result has not been described in previous studies. A previous study with a relatively small sample size (*n* = 4,875) reported comparable complication rates between LCC and RCC patients undergoing colectomy with the exception of superficial SSI, which was found to be less common in RCC colectomy [33]. Reasons for this difference may be associated with a delay in the diagnosis and associated advanced stage of RCC [34–36]. Additionally, the occurrence rate of mild and severe anemia was lower in LCC than in RCC patients. We suspect that LCC patients exhibited a better physical condition and earlier presentation than RCC patients.

Our study had several limitations related to its retrospective design. Although the NSQIP is a large worldwide database, the samples included in our study are likely heterogeneous and subject to selection bias because the patients were all deemed fit for surgery. We were unable to consider the specific therapy for each patient. Additionally, the NSQIP does not provide detailed data on the subtype of anemia, duration prior to surgery, volume of blood transfusion, and estimated blood loss during the operation. Sorting these factors out of such a large database would be impractical. Finally, the database does not define the transfusion criteria or rationale.

## Conclusions

The study found that perioperative blood transfusion posed a greater risk of complications and mortality regardless of anemia status, suggesting that a perioperative blood transfusion should be judiciously administered, particularly in patients with mild anemia or without anemia.

## Authors’ contributions

Z.L. and J.J.L. contributed to the study concept and design; acquisition, analysis, and interpretation of the data; and drafting of the manuscript. K.Y.P., S.A.K, X.X.W., Z.X.Z., M.Y., C.H.J., X.S.W., and Y.W.Z. contributed to the study concept and design, analysis, and interpretation of the data, and critical revision of the manuscript for important intellectual content. All authors read and approved the final manuscript.

## Funding

This work was supported by the Beijing Municipal Science & Technology Commission [No. Z161100000116090], the National Key Research and Development Program of the Ministry of Science and Technology of China [No. 2016YFC0905303], the CAMS Innovation Fund for Medical Sciences (CIFMS) [No.2016-I2M-1–001], and the Beijing Science and Technology Program [No. D17110002617004].

## Supplementary Material

goz033_Supplementary_Data
