# Supplementary material for: Joint effect of pre-operative anemia and perioperative blood transfusion on outcomes of colon-cancer patients undergoing colectomy
Source: Gastroenterol Rep (Oxf). 2019 Aug 9;8(2):151–7. doi: 10.1093/gastro/goz033 (PMC7136710; doi:10.1093/gastro/goz033)
Supplement: goz033_Supplementary_Data [file goz033_supplementary_data.docx]

Table S1. Association between surgical outcomes and operation approach by anemia and blood transfusion

Abbreviations:

OR, odds ratio.

Footnote:

^a^ Adjusted for age, sex, race, smoking, functional status, American Society of Anesthesiologists classification, body mass index, weight loss>10%, diabetes, congestive heart failure, previous cardiac surgery, previous percutaneous coronary intervention, myocardial infraction, transient ischemic attack, hypertension, pneumonia, chronic obstructive pulmonary disease, cerebrovascular accident, and hemiplegia.

^b^ Including at least 1 or more complications.

^c^ Including superficial surgical site infection, deep vein thrombosis, urinary tract infection and/or thrombophlebitis.

^d^ Including deep surgical site infection, organ-space surgical site infection, wound disruption, pneumonia, reintubation, pulmonary embolism, greater than 48-hour postoperative ventilator-assisted respiration, progressive renal insufficiency, acute renal failure, cerebrovascular accident, cardiac arrest requiring cardiopulmonary resuscitation, myocardial infraction, sepsis, and septic shock.

| Variable | No complications | Any complication^b^ | | Minor complications^c^ | | Major complications^d^ | | 30-day mortality | |
| --- | --- | --- | --- | --- | --- | --- | --- | --- | --- |
|  | N | N | OR^a^ (95% CI) | N | OR^a^ (95% CI) | N | OR^a^ (95% CI) | N | OR^a^ (95% CI) |
| Open |  |  |  |  |  |  |  |  |  |
| Anemia |  |  |  |  |  |  |  |  |  |
| No | 4,836 | 1,038 | 1 [Reference] | 581 | 1 [Reference] | 539 | 1 [Reference] | 61 | 1 [Reference] |
| Mild | 6,387 | 1,871 | 1.15 (1.05-1.26) | 980 | 1.11 (0.98-1.25) | 1,059 | 1.19 (1.05-1.34) | 200 | 1.53 (1.12-2.09) |
| Severe | 309 | 87 | 0.89 (0.68-1.15) | 46 | 0.92 (0.66-1.29) | 54 | 0.91 (0.66-1.26) | 10 | 1.31 (0.65-2.67) |
| Transfusion |  |  |  |  |  |  |  |  |  |
| No | 10,604 | 2,505 | 1 [Reference] | 1,389 | 1 [Reference] | 1,316 | 1 [Reference] | 213 | 1 [Reference] |
| Yes | 928 | 491 | 2.10 (1.86-2.38) | 218 | 1.73 (1.47-2.05) | 336 | 2.70 (2.33-3.13) | 58 | 1.83 (1.34-2.50) |
| Anemia/transfusion |  |  |  |  |  |  |  |  |  |
| No anemia, no transfusion | 4,749 | 979 | 1 [Reference] | 557 | 1 [Reference] | 494 | 1 [Reference] | 57 | 1 [Reference] |
| No anemia, transfusion | 87 | 59 | 3.44 (2.44-4.84) | 24 | 2.50 (1.57-3.98) | 45 | 5.27 (3.61-7.69) | 4 | 2.39 (0.85-6.77) |
| Mild anemia, no transfusion | 5,659 | 1,485 | 1.18 (1.08-1.30) | 804 | 1.12 (0.99-1.27) | 801 | 1.26 (1.11-1.43) | 149 | 1.54 (1.11-2.13) |
| Mild anemia, transfusion | 728 | 386 | 2.32 (2.00-2.69) | 176 | 1.90 (1.57-2.31) | 258 | 3.05 (2.55-3.65) | 51 | 2.95 (1.97-4.42) |
| Severe anemia, no transfusion | 196 | 41 | 0.94 (0.66-1.33) | 28 | 1.12 (0.74-1.69) | 21 | 0.92 (0.58-1.47) | 7 | 2.15 (0.95-4.86) |
| Severe anemia, transfusion | 113 | 46 | 1.83 (1.28-2.61) | 18 | 1.27 (0.77-2.12) | 33 | 2.58 (1.71-3.87) | 3 | 1.28 (0.39-4.18) |
| *P* value for interaction |  |  | <0.001 |  | 0.02 |  | <0.001 |  | 0.05 |
| Laparoscopic |  |  |  |  |  |  |  |  |  |
| Anemia |  |  |  |  |  |  |  |  |  |
| No | 10,000 | 1,028 | 1 [Reference] | 581 | 1 [Reference] | 510 | 1 [Reference] | 45 | 1 [Reference] |
| Mild | 8,719 | 1,231 | 1.17 (1.06-1.28) | 591 | 1.05 (1.05-1.05) | 718 | 1.30 (1.29-1.30) | 103 | 1.26 (0.86-1.85) |
| Severe | 302 | 55 | 1.13 (0.82-1.54) | 24 | 1.12 (1.11-1.14) | 42 | 1.47 (1.46-1.48) | 7 | 1.22 (0.50-2.93) |
| Transfusion |  |  |  |  |  |  |  |  |  |
| No | 18,041 | 2,062 |  | 1,115 | 1 [Reference] | 1,085 | 1 [Reference] | 119 | 1 [Reference] |
| Yes | 980 | 252 | 1.93 (1.65-2.25) | 81 | 1.26 (1.25-1.27) | 185 | 2.67 (2.66-2.68) | 36 | 3.19 (2.11-4.82) |
| Anemia/transfusion |  |  |  |  |  |  |  |  |  |
| No anemia, no transfusion | 9,885 | 989 | 1 [Reference] | 568 | 1 [Reference] | 482 | 1 [Reference] | 37 | 1 [Reference] |
| No anemia, transfusion | 115 | 39 | 3.28 (2.25-4.77) | 13 | 2.05 (2.02-2.08) | 28 | 5.39 (5.33-5.44) | 8 | 12.17 (5.39-27.49) |
| Mild anemia, no transfusion | 7,986 | 1,041 | 1.19 (1.08-1.31) | 530 | 1.06 (1.06-1.07) | 578 | 1.33 (1.33-1.33) | 78 | 1.51 (1.00-2.29) |
| Mild anemia, transfusion | 733 | 190 | 2.22 (1.85-2.66) | 61 | 1.32 (1.31-1.33) | 140 | 3.50 (3.49-3.52) | 25 | 4.03 (2.35-6.90) |
| Severe anemia, no transfusion | 170 | 32 | 1.69 (1.14-2.49) | 17 | 1.64 (1.62-1.66) | 25 | 2.72 (2.70-2.75) | 4 | 2.90 (0.98-8.53) |
| Severe anemia, transfusion | 132 | 23 | 1.46 (0.92-2.30) | 7 | 0.80 (0.78-0.81) | 17 | 2.19 (2.16-2.21) | 3 | 2.58 (0.76-8.76) |
| *P* value for interaction |  |  | <0.001 |  | <0.001 |  | <0.001 |  | <0.001 |

Table S2 Association between surgical outcomes and length of surgery by anemia and blood transfusion

Abbreviations:

OR, odds ratio.

Footnote:

^a^ Adjusted for age, sex, race, smoking, functional status, American Society of Anesthesiologists classification, body mass index, weight loss>10%, diabetes, congestive heart failure, previous cardiac surgery, previous percutaneous coronary intervention, myocardial infraction,transient ischemic attack, hypertension, pneumonia, chronic obstructive pulmonary disease, cerebrovascular accident, and hemiplegia.

^b^ Including at least 1 or more complications.

^c^ Including superficial surgical site infection, deep vein thrombosis, urinary tract infection and/or thrombophlebitis.

^d^ Including deep surgical site infection, organ-space surgical site infection, wound disruption, pneumonia, reintubation, pulmonary embolism, greater than 48-hour postoperative ventilator-assisted respiration, progressive renal insufficiency, acute renal failure, cerebrovascular accident, cardiac arrest requiring cardiopulmonary resuscitation, myocardial infraction, sepsis, and septic shock.

|  | No complications | Any complication^b^ | | Minor complications^c^ | | Major complications^d^ | | 30-day mortality | |
| --- | --- | --- | --- | --- | --- | --- | --- | --- | --- |
|  | N | N | OR^a^ (95% CI) | N | OR^a^ (95% CI) | N | OR^a^ (95% CI) | N | OR^a^ (95% CI) |
| Length of surgery <160 minutes |  |  |  |  |  |  |  |  |  |
| Anemia |  |  |  |  |  |  |  |  |  |
| No | 8,221 | 1,019 | 1 [Reference] | 567 | 1 [Reference] | 505 | 1 [Reference] | 56 | 1 [Reference] |
| Mild | 9,528 | 1,830 | 1.24 (1.14-1.36) | 635 | 1.20 (1.07-1.35) | 771 | 1.30 (1.15-1.46) | 111 | 1.49 (1.08-2.06) |
| Severe | 391 | 73 | 0.94 (0.71-1.23) | 29 | 1.08 (0.76-1.53) | 54 | 0.92 (0.65-1.31) | 5 | 1.64 (0.83-3.23) |
| Transfusion |  |  |  |  |  |  |  |  |  |
| No | 17,095 | 2,571 | 1 [Reference] | 1,106 | 1 [Reference] | 1,082 | 1 [Reference] | 119 | 1 [Reference] |
| Yes | 1,045 | 351 | 1.90 (1.66-2.18) | 153 | 1.51 (1.25-1.82) | 287 | 2.41 (2.05-2.84) | 47 | 1.89 (1.34-2.66) |
| Anemia × transfusion |  |  |  |  |  |  |  |  |  |
| No anemia, no transfusion | 8,128 | 981 | 1 [Reference] | 553 | 1 [Reference] | 477 | 1 [Reference] | 52 | 1 [Reference] |
| No anemia, transfusion | 93 | 38 | 3.44 (2.33-5.09) | 14 | 2.31 (1.30-4.10) | 28 | 5.27 (3.38-8.20) | 4 | 4.26 (1.49-12.18) |
| Mild anemia, no transfusion | 8,723 | 1,547 | 1.27 (1.16-1.39) | 816 | 1.21 (1.07-1.36) | 819 | 1.35 (1.19-1.53) | 152 | 1.54 (1.10-2.15) |
| Mild anemia, transfusion | 805 | 283 | 2.34 (1.99-2.74) | 120 | 1.86 (1.50-2.32) | 187 | 3.06 (2.52-3.72) | 40 | 3.06 (1.97-4.77) |
| Severe anemia, no transfusion | 244 | 43 | 1.21 (0.86-1.70) | 29 | 1.47 (0.99-2.20) | 23 | 1.27 (0.81-1.98) | 9 | 3.01 (1.43-6.32) |
| Severe anemia, transfusion | 147 | 30 | 1.34 (0.90-2.02) | 12 | 0.99 (0.54-1.81) | 19 | 1.69 (1.03-2.78) | 3 | 1.38 (0.42-4.55) |
| *P* value for interaction |  |  | <0.001 |  | 0.003 |  | <0.001 |  | 0.008 |
| Length of surgery >160 minutes |  |  |  |  |  |  |  |  |  |
| Anemia |  |  |  |  |  |  |  |  |  |
| No | 6,615 | 1,047 | 1 [Reference] | 595 | 1 [Reference] | 544 | 1 [Reference] | 50 | 1 [Reference] |
| Mild | 5,578 | 1,272 | 1.19 (1.08-1.31) | 635 | 1.09 (0.96-1.24) | 771 | 1.32 (1.16-1.50) | 111 | 1.49 (1.03-2.15) |
| Severe | 220 | 69 | 1.20 (0.89-1.62) | 29 | 1.05 (0.70-1.60) | 54 | 1.57 (1.12-2.21) | 5 | 1.02 (0.38-2.72) |
| Transfusion |  |  |  |  |  |  |  |  |  |
| No | 11,550 | 1,996 | 1 [Reference] | 1,106 | 1 [Reference] | 1,082 | 1 [Reference] | 119 | 1 [Reference] |
| Yes | 863 | 392 | 2.32 (2.02-2.66) | 153 | 1.73 (1.42-2.10) | 287 | 2.92 (2.49-3.43) | 47 | 3.07 (2.11-4.46) |
| Anemia × transfusion |  |  |  |  |  |  |  |  |  |
| No anemia, no transfusion | 6,506 | 987 | 1 [Reference] | 572 | 1 [Reference] | 499 | 1 [Reference] | 42 | 1 [Reference] |
| No anemia, transfusion | 109 | 60 | 3.57 (2.57-4.95) | 23 | 2.43 (1.53-3.86) | 45 | 5.24 (3.64-7.56) | 8 | 7.37 (3.36-16.16) |
| Mild anemia, no transfusion | 4,922 | 979 | 1.22 (1.10-1.35) | 518 | 1.11 (0.97-1.26) | 560 | 1.39 (1.21-1.59) | 75 | 1.72 (1.16-2.56) |
| Mild anemia, transfusion | 656 | 293 | 2.66 (2.26-3.13) | 117 | 1.87 (1.49-2.34) | 211 | 3.74 (3.09-4.53) | 36 | 4.56 (2.84-7.34) |
| Severe anemia, no transfusion | 122 | 30 | 1.52 (1.01-2.29) | 16 | 1.42 (0.83-2.41) | 23 | 2.29 (1.44-3.64) | 2 | 1.78 (0.42-7.54) |
| Severe anemia, transfusion | 98 | 39 | 2.39 (1.63-3.52) | 13 | 1.38 (0.76-2.49) | 31 | 3.73 (2.44-5.71) | 3 | 2.73 (0.81-9.17) |
| *P* value for interaction |  |  | 0.002 |  | 0.04 |  | <0.001 |  | 0.007 |

Table S3 Goodness of fit for logistic models in this study

Abbreviations:

LCC, left colon cancer; RCC, right colon cancer; AUC, area under curve.

Footnote:

^a^ Including at least 1 or more complications.

^b^ Including superficial surgical site infection, deep vein thrombosis, urinary tract infection and/or thrombophlebitis.

^c^ Including deep surgical site infection, organ-space surgical site infection, wound disruption, pneumonia, reintubation, pulmonary embolism, greater than 48-hour postoperative ventilator-assisted respiration, progressive renal insufficiency, acute renal failure, cerebrovascular accident, cardiac arrest requiring cardiopulmonary resuscitation, myocardial infraction, sepsis, and septic shock.

|  | Any complication^a^ | | Minor complications^b^ | | Major complications^c^ | | 30-day mortality | |
| --- | --- | --- | --- | --- | --- | --- | --- | --- |
|  | AUC | Hosmer-Lemeshow  *P* value | AUC | Hosmer-Lemeshow  *P* value | AUC | Hosmer-Lemeshow  *P* value | AUC | Hosmer-Lemeshow  *P* value |
| LCC |  |  |  |  |  |  |  |  |
| Anemia | 0.735 | 0.281 | 0.736 | 0.437 | 0.758 | 0.803 | 0.805 | 0.863 |
| Transfusion | 0.744 | 0.554 | 0.736 | 0.829 | 0.770 | 0.437 | 0.815 | 0.809 |
| Anemia and Transfusion | 0.749 | 0.628 | 0.740 | 0.468 | 0.779 | 0.595 | 0.820 | 0.842 |
| RCC |  |  |  |  |  |  |  |  |
| Anemia | 0.709 | 0.310 | 0.700 | 0.549 | 0.736 | 0.498 | 0.738 | 0.530 |
| Transfusion | 0.723 | 0.263 | 0.706 | 0.745 | 0.757 | 0.155 | 0.742 | 0.610 |
| Anemia and Transfusion | 0.726 | 0.257 | 0.707 | 0.371 | 0.760 | 0.135 | 0.753 | 0.428 |
